# Development of colonic transit time and ultrasound imaging tools as objective indicators for assessing abnormal defecation associated with food intake: a narrative review based on previous scientific knowledge

**DOI:** 10.1186/s13030-021-00222-1

**Published:** 2021-11-06

**Authors:** Yohei Okawa

**Affiliations:** Department of Behavioural Medicine, Tohoku University Graduate School of Medicine, 2-1 Seiryo-machi, Aoba-ku, Sendai, Miyagi 980-8575 Japan

**Keywords:** Functional gastrointestinal disorders, FGIDs, Irritable bowel syndrome, IBS, Functional constipation, FC, Irritable bowel syndrome with constipation, IBS-C, Normal transit constipation, NTC, Slow transit constipation, STC

## Abstract

**Background:**

Functional gastrointestinal disorders (FGIDs) involve chronic or persistent gastrointestinal symptoms. Laboratory tests show no organic lesions, and the symptoms are due to dysfunction. The most typical FGID is irritable bowel syndrome (IBS). In IBS patients, defecation disorders are common and have adverse effects on daily life. The proper evaluation and analysis of colonic transit are important for the management of defecation disorders in IBS patients. In addition, dietary intake and lifestyle affect colonic transit. An accurate assessment of such factors can guide management, leading to improvements in colonic transit and the resolution of defecation disorders.

**Main topic:**

The Rome IV diagnostic criteria for IBS are based on subjective symptoms, which must be communicated and explained by the patient, limiting their application. Colonic transit time and ultrasonography are objective tools that can be used to diagnose IBS. In particular, previous studies used colonic transit to accurately distinguish between constipation and normal stool passage and to assess delayed gastrointestinal motility. Diet and lifestyle modifications can improve colonic transit and ameliorate bowel dysfunction.

**Conclusion:**

Colonic transit can be improved by modifying lifestyle factors. Defecation disorders in IBS patients may be resolved by focusing on such factors. In the future, methods of visualizing defecation disorders due to impaired gastrointestinal motility and objective indicators of the associated abdominal symptoms need to be investigated.

## Background

### Overview of functional gastrointestinal disorders

Functional gastrointestinal disorders (FGIDs) are a group of diseases characterized by chronic gastrointestinal symptoms despite the absence of organic abnormalities in the gastrointestinal tract. Many patients with FGIDs also have psychosomatic pathology, suggesting a connection between the gastrointestinal tract and brain via the gut-brain axis [1, 2]. Typical FGIDs include functional dyspepsia (FD), irritable bowel syndrome (IBS), and functional constipation. In particular, the typical characteristics of IBS are chronic and relapsing abdominal pain and discomfort associated with abnormal bowel movements [1–3].

### IBS diagnostic criteria

The Rome IV diagnostic criteria, which are based on subjective symptoms, are used to diagnose IBS [1–4] and can distinguish between chronic symptoms of functional bowel disease and transient gastrointestinal symptoms. The criteria are the presence of abdominal pain at least 1 day per week in the past 3 months associated with at least two of the following: pain associated with defecation, changes in defecation frequency, and changes in stool appearance. According to these diagnostic criteria, functional bowel disease can be diagnosed when the symptoms have existed for longer than 6 months before diagnosis and have occurred on at least 1 day per week for the past 3 months [1–4]. IBS subtypes include IBS with diarrhoea (IBS-D), IBS with constipation (IBS-C), mixed IBS (IBS-M), and unclassifiable IBS (IBS-U); these subtypes are believed to be useful in clinical practice and treatment. It is difficult to diagnose patients who are unconscious or have cognitive impairment because the Rome IV criteria evaluate only subjective symptoms. In addition, the stool in patients with IBS varies from watery to hard, suggesting that the time stool takes to pass through the gastrointestinal tract, reflected in its shape, also varies [5, 6]. It has also been reported that defecation frequency and transit time are often the same in IBS patients and healthy subjects [7, 8]. IBS causes bowel dyskinesias such as constipation, diarrhoea, and abnormal bowel movements, but the symptoms related to defecation vary by subtype. Therefore, IBS is a clear risk factor for faecal incontinence [9–11].

## Main topics

### Methods

This paper suggests that dietary therapy improves colonic transit time in patients with chronic constipation and cures defecation disorders. It is a review of the current literature, presents the existing knowledge, and discusses future prospects. The articles selected were published from 1983 to 2020, and the discourse is based on articles with scientific knowledge for which consensus has already been obtained. Papers were retrieved from PubMed using the keywords “Colonic Transit”, “Functional Gastrointestinal Disorders, FGIDs”, “Irritable Bowel Syndrome, IBS”, and “Dietary Therapy”. The search was limited to published studies without restrictions on date, study design, or patient age.

### Mechanisms and symptoms of gastrointestinal motility

It has been reported that the onset of IBS symptoms is related to symptom recognition and the perception of internal organs in the gastrointestinal tract by the brain; in recent years, a correlation between cerebral and intestinal function has been shown [12–14]. The gut-brain axis is a functional association between the central nervous system and the gastrointestinal tract and is associated with the development of visceral hypersensitivity that causes abdominal pain and discomfort. In the pathway leading to the perception of visceral organs by the brain, a stimulus from the digestive tract is first transmitted to the sensory neurons of the spinal nerve and then transmitted to the visceral sensory neurons in the posterior horn of the spinal cord via the dorsal root. The signal ascends to the thalamus, from which it is projected to the neuronal islands, anterior cingulate gyrus, and prefrontal cortex, resulting in perception of the visceral stimulus [12]. Hypersensitivity has been shown to occur when the intensity of this stimulus is high or when the threshold for the perception of a visceral stimulus is reduced [13, 14]. Especially in IBS, the threshold for the perception of sensations in the gastrointestinal tract is reduced, suggesting that hyperesthesia is more likely to occur in patients with IBS than in healthy subjects [12].

In addition, gastrointestinal dyskinesia and visceral hypersensitivity are associated with abdominal pain and discomfort in patients with IBS [1, 2]. Previous studies have shown correlations between colonic transit time in patients with IBS-C and abdominal pain and bloating [6]. In addition, defecation frequency and the degree of stool looseness are negatively correlated with gastrointestinal transit time, and it is important to evaluate gastrointestinal transit time in patients with IBS-C [13]. Thus, it is important to treat the visceral hypersensitivity and gastrointestinal motility abnormalities that cause abdominal pain and abdominal discomfort in IBS, but such treatments have not yet been developed.

FC constitutes a group of functional and chronic diseases without identifiable organic lesions in the lower gastrointestinal tract. Unlike IBS-C, FC is defined by the absence of abdominal pain or discomfort but the presence of a high frequency of bowel movements [3]. In recent years, colon function in patients with FGIDs has been clinically evaluated by measuring gastrointestinal transit time using opaque markers on abdominal X-ray [6]. It is possible to evaluate gastrointestinal function by evaluating the quantity and location of residual markers in the gastrointestinal tract on abdominal radiography [15, 16]. Furthermore, this method can be used to differentiate constipation based on colon transit time into normal-transit constipation (NTC) (less than 3 days) and slow-transit constipation (STC) (3 days or more). It has been reported that pelvic floor dysfunction can also be detected using this method [15, 17, 18].

In a previous study, gastrointestinal transit time was measured in 359 IBS patients: 287 (80%) had a normal transit time, 19 (5%) had a delayed transit time, and 53 (15%) had an accelerated transit time. Furthermore, a comparison of gastrointestinal transit time among patients with different IBS subtypes, namely, IBS-C, IBS-D, and IBS-M, as defined based on the Rome III diagnostic criteria, showed that 15% of the IBS-C patients had delayed gastrointestinal transit, 36% of the IBS-D patients had accelerated gastrointestinal transit, and some IBS patients had a normal transit time [6]. Patients with each IBS subtype were found to have normal gastrointestinal transit, and delayed gastrointestinal transit was identified in 15% of the patients with IBS-C; these findings suggest that gastrointestinal motility is impaired in patients with IBS-C but not in patients with other IBS subtypes [6]. In addition, previous studies have compared gastrointestinal transit time between patients with IBS-C and FC and shown no significant difference [19]. IBS-C is characterized by more bloating and abdominal pain than FC, and IBS symptoms such as abdominal pain and defecation disorders have been shown to be correlated with gastrointestinal transit time [6, 19].

As mentioned above, few studies have reported objective indicators for diagnosing IBS, which would be useful for assessing IBS patients who are unconscious. Such patients cannot make decisions based on Rome criteria. Therefore, I think that an objective index is necessary for IBS diagnosis.

Previous studies from various perspectives have evaluated colonic transit time [20–22]. By examining gastrointestinal transit time, it is possible to determine the presence or absence of abnormal gastrointestinal motility, which may provide an indicator of the IBS subtype.

However, there are also critical issues; for example, gastrointestinal transit time may not correlate with IBS with sufficient detection sensitivity [6, 19]. However, non-invasive tests may be able to classify IBS subtypes; in particular, IBS subtypes can be assessed by measuring gastrointestinal transit time, which is expected to be useful for diagnosis.

In addition, previous studies have reported lower gastrointestinal thresholds in IBS patients. Compared to healthy subjects, IBS patients have greater gastrointestinal perception, and decreased rectal compliance and increased internal pressure have been reported [23]. Subjective indicators alone cannot be used for a detailed diagnostic assessment of IBS in unconscious patients, for whom objective indicators are necessary. Further research evaluating gastrointestinal motility in IBS patients is necessary.

### Faecal drainage disorder associated with rectal hyposensitivity and functional defecation

**Fig. 1:**
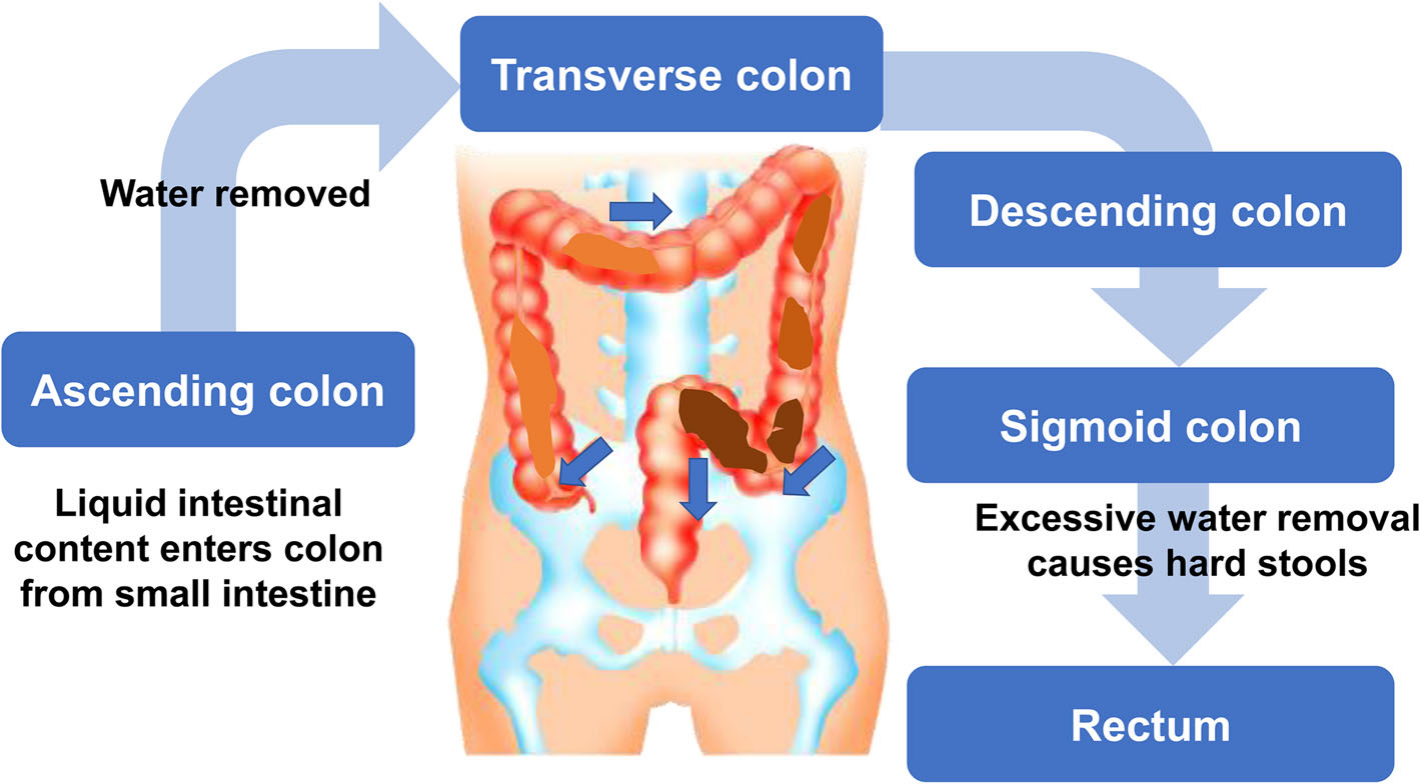
Defecation mechanism. In normal defecation, the lack of an urge to defecate indicates the absence of stool in the rectum. In this case, faeces are stored on the oral side of the sigmoid colon. There are two causes of dysfunction: rectal hypoesthesia and functional stool drainage disorders. Measuring the gastrointestinal transit time is useful for evaluating these causes of dysfunction

In the case of normal defecation function, when there is no stool in the rectum, there is no urge to defecate. Faeces are stored on the oral side of the sigmoid colon (Fig. 1). When substantial peristalsis occurs in the left hemicolon, faeces stored in the sigmoid colon immediately move to the rectum, and the intestinal wall is stretched. This stretching creates a signal that is transmitted to the cerebral cortex via the sacral nerve, and the urge to defecate is felt. When the rectal wall is stretched by faeces, the transient external anal sphincter contracts, and the oral side of the internal anal sphincter relaxes. This is the rectal-anal inhibitory reflex, and a portion of the rectal content reaches the vicinity of the dentate line. Unlike the rectal mucosa, the dentate line has sensation, enabling perception of the properties of the rectal contents (solid stool, liquid stool, gas, etc.). In a situation suitable for defecation, an individual can assume the appropriate posture (leaning forward) and push (to increase the intra-abdominal pressure). At the same time, the external anal sphincter and puborectalis muscles relax, allowing defecation [24–27].

When any of these rectal-anal functions is impaired, a “faecal drainage disorder” occurs in the rectum, and faeces cannot be comfortably discharged. There are two major stool discharge disorders, rectal hyposensitivity and functional defecation disorder [28–30]. Rectal hyposensitivity is a condition in which rectal sensation is reduced, and the presence of stool cannot be detected. Functional defecation disorder is a condition in which faeces in the rectum cannot be comfortably discharged because the coordinated movements of “straining” and relaxation of the pelvic floor muscles are not achieved during defecation. It is important to analyse whether patients with these stool discharge disorders have normal or abnormal function and to understand their pathological condition.

### Faecal discharge disorders can be assessed by colonic transit time

Colonic transit time is a test that objectively evaluates peristaltic movement of the large intestine. As mentioned above, both NTC and STC involve pelvic floor dysfunction [15, 17, 18]. To distinguish between NTC and STC, as a general rule, all drugs that affect defecation, such as laxatives, are discontinued before testing. Colonic transit study methods include opaque X-ray markers such as SITZMARKS®, scintigraphy using radioisotopes, and wireless testing using a Smart Pill® to measure the internal pressure, temperature, and pH in the intestinal tract. The marker method requires 5 to 7 days to complete the test, whereas scintigraphy can be performed in 24 to 48 h, and the correlation between the results of the two methods is good [31]. However, the scintigraphy method is used only in research because it requires the use of radioisotopes and is thus not widely used in clinical practice. The results of the wireless capsule method also correlate well with those of the marker method [32]. Although this method provides more detailed gastrointestinal motility data, its clinical usefulness has not been established [18]; it has not been used in studies in Japan.

The clinical significance of the colon transit time test has been established, and the marker method is the most widespread worldwide [18]. However, although the marker method is generally considered a colon transit time test, it actually evaluates the time from marker ingestion to excretion from the anus. Therefore, it indicates the transit time through the entire gastrointestinal tract. An assumption of no significant dysfunction in the upper gastrointestinal tract is made when the marker method is used as a colon transit time test.

**Fig. 2:**
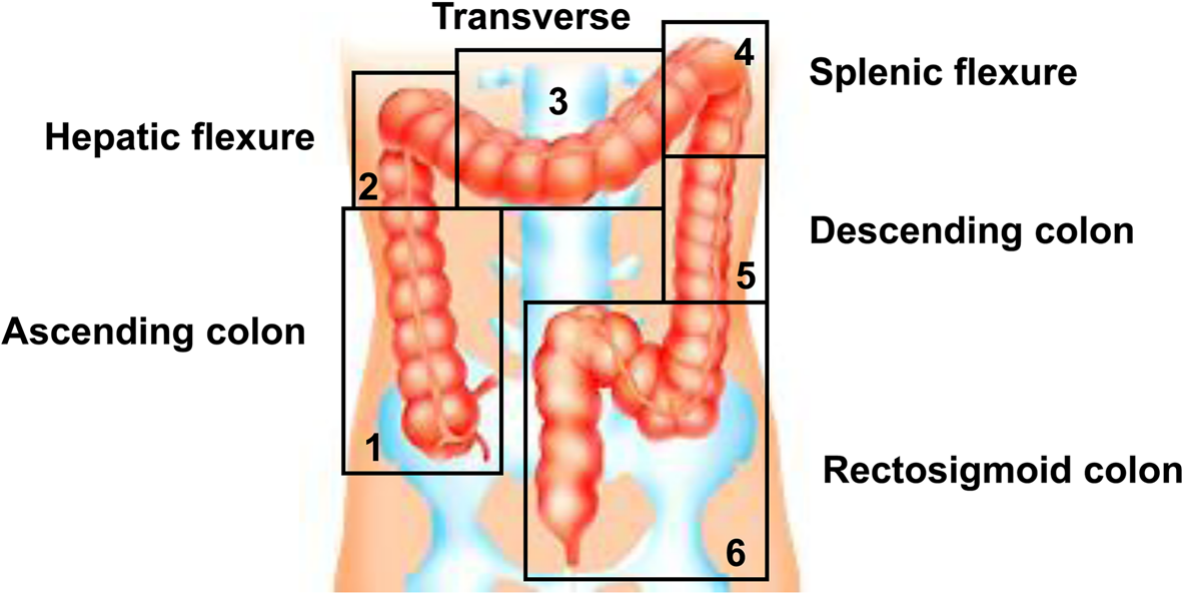
Classification of opaque marker retention in the large intestine on X-ray. A method has been developed to divide the large intestine into the right colon, left colon, sigmoid colon, and rectum and to classify disorders into total colon stagnation, right colon stagnation, left colon stagnation, and faecal discharge disorder according to marker retention at each site

In addition, SITZMARKS®, which is used in the marker method, has not yet been approved by regulatory agencies nor is it covered by insurance in Japan. Therefore, it is possible for a doctor to import a marker from a foreign country into Japan and use it; however, it can be used in only medical care paid for out-of-pocket or clinical research approved by an ethics committee. The marker method was developed in 1969, and standard values have been established for healthy subjects [33]. With regard to SITZMARKS®, it has been shown that individuals “normally excrete 80% or more of the marker 5 days after oral administration of the marker” [30, 34]. Various additional methods have been developed, and the standard values vary. Furthermore, a method has been devised of dividing the large intestine into the right colon, left colon, sigmoid colon, and rectum and classifying the results into total colon stagnation, right colon stagnation, left colon stagnation, and stool discharge disorder according to marker retention at each site [35, 36] (Fig. 2). Although these methods are useful for evaluating patient pathology, their clinical significance has not yet been established. Additional study will be necessary to validate the clinical usefulness of these methods.

### Do psychosocial factors affect colonic transit time?

IBS is also a psychological disorder; specifically, the severity of IBS is associated with psychological abnormalities [20], including depression and anxiety, which are accompanied by physical symptoms [20, 37]. Previous studies have shown that such psychological abnormalities are a risk factor for the development of IBS [38].

Stress is also involved in IBS development. Previous studies have reported that gastrointestinal symptoms associated with IBS are exacerbated by stress and that there is a correlation between stress load and worsening gastrointestinal symptoms [39]. In addition, it has been reported that IBS patients have increased colorectal motility and colorectal smooth muscle electromyography as measured by internal pressure of the large intestine when psychosocial stress occurs [40, 41]. Therefore, psychosocial factors may be involved in exacerbating IBS symptoms and abnormal gastrointestinal motility. However, no studies have decisively linked gastrointestinal transit time with psychosocial factors. Further research will be necessary to clarify which psychosocial factors are associated with transit time.

### Does colonic transit improve after modifying food intake and lifestyle factors?

People with IBS often have normal social lives, and their daily living habits may affect the symptoms of defecation disorders. Previous studies have shown that IBS-associated abdominal pain and discomfort are related to food intake and that certain foods exacerbate the symptoms [42–45], in particular foods high in carbohydrates and fats, coffee, alcohol, and spices [42, 43, 46]. Dietary fibre contained in food, including water-soluble and insoluble dietary fibre, is also important. The consumption of water-soluble dietary fibre has been reported to lead to an improvement in IBS symptoms such as abdominal pain and defecation disorders [47, 48]. In a study comparing the effects of water-soluble dietary fibre and insoluble dietary fibre consumption on IBS symptoms, the consumption of water-soluble dietary fibre led to a significant improvement in IBS symptoms, while the consumption of insoluble dietary fibre did not. Cases of exacerbation have been reported [47, 48]. In particular, the consumption of the dietary fibre source psyllium (Plantainaceae) has been shown to be effective at reducing IBS symptoms [47]. Psyllium is the powdered seed coat of species in the Plantago genus and contains a large amount of dietary fibre [46]. Previous studies on IBS have shown that psyllium consumption significantly improved IBS symptoms after 12 weeks compared to consumption of bran and a placebo [48]. In addition, the results of a randomized controlled trial of FC showed that 4 weeks of psyllium intake significantly increased the values for complete spontaneous defecation (CSBM) and the Bristol stool shape scale (BSFS), and the force during defecation (straining) and bloating were significantly reduced. Thus, previous studies on food intake suggest that psyllium intake is effective for the treatment of FC and IBS [49].

There have been various reports on the relation between food intake and constipation symptoms. In particular, psyllium has been found in recent years to improve abdominal pain, abdominal discomfort, and bowel movement abnormalities in IBS patients and to be effective for the treatment of bowel movement abnormalities in patients with FC.

### Foods that affect gastrointestinal motility

More recently, it has been shown that the intake of certain carbohydrates causes symptoms. Foods high in FODMAPs, a generic term for fermentable oligosaccharides, disaccharides, monosaccharaides, and polyols, have been shown to cause IBS-related abdominal pain and discomfort [46, 50–52]; such foods include clingstone peaches, apricots, chickpeas and lentils, whereas kiwifruit, blueberry, grapefruit, and honeydew melon are low-FODMAP foods [46].

FODMAPs such as oligosaccharides and polyols, including lactose, fructose, fructans, and galactans, are not easily absorbed in the small intestine, and fermentation is promoted in the large intestine. Fermentation of these indigestible carbohydrates produces short-chain fatty acids, the general term for acetic acid, propionic acid, butyric acid, isobutyric acid, lactic acid, and succinic acid, in the large intestine. Short-chain fatty acid production occurs when carbohydrates are fermented by enterobacteria such as *Lactobacillus* and *Veillonella*, which are more abundant in patients with IBS than in healthy subjects, suggesting that the short-chain fatty acids produced by these bacteria cause abdominal pain and discomfort [46]. A low-FODMAP diet has been shown to be effective in several clinical trials [50–52]. A randomized controlled trial showed that a low-FODMAP diet significantly improved IBS symptoms compared to a conventional Australian diet [51]. In addition, a randomized controlled trial comparing a low-FODMAP diet with a high-FODMAP diet reported that a low-FODMAP diet significantly reduced the intensity and frequency of abdominal pain and disease severity in patients with IBS [52]. In addition, a meta-analysis of four clinical studies on low-FODMAP diets showed the effectiveness of low-FODMAP diets at improving IBS symptoms, although each study had different dietary contents, observation periods, and endpoints [50]. However, individuals on low-FODMAP diets refrain from ingesting certain non-absorbable sugars, and there are concerns about nutritional bias and adverse effects on intestinal bacteria [46, 53].

### Can IBS be evaluated objectively and noninvasively by methods other than colonic transit measurements?

There are few epidemiological studies on the usefulness of non-colon endoscopy for the diagnosis of IBS, although some studies have investigated ultrasonography and upper gastrointestinal endoscopy [54]. Previous studies on ultrasound reported that gallbladder contraction was higher in the IBS group than in the control group, both in the fasted state and after dietary stress [55, 56]. Although IBS has been shown to be associated with cholecystectomy, a direct association remains unclear due to the small number of cases. In addition, a previous study in Japan reported the use of abdominal ultrasonography to assess colon motility, and observation of the sigmoid colon on an empty stomach showed enhanced colonic contraction in an IBS group. Postprandial observations of the sigmoid colon were compared between nine IBS patients diagnosed by the Rome II criteria and four controls. Segmentation was enhanced in patients with IBS-C, but patients with IBS-D had enhanced transport of intestinal contents to the anus [57]. Based on these reports, ultrasonography can be considered a useful non-invasive test and is expected to have clinical utility for assessing bowel motility. However, because there have been few reports, future verification is needed.

In addition, there are limited reports of the use of ultrasound to diagnose IBS. Among the existing reports, some confirmed the characteristic changes in the gastrointestinal tract of IBS patients on ultrasound. A previous study used ultrasound to investigate the gastric emptying rate (GER) and sinus motility in 76 children with IBS who met the Rome III diagnostic criteria. The GER was significantly reduced in the IBS group after stress, showing that stress more seriously impairs the GER and sinus movement in patients with IBS than in healthy people. On ultrasound, the GER and pyloric movement indicated delayed gastric emptying in children with all four IBS subtypes in addition to impaired pyloric sinus movement. However, no clear relation between gastrointestinal dyskinesia and symptoms has been shown [58]. A previous study investigating whether transvaginal ultrasound was useful for IBS diagnosis reported thickening of the intestinal wall of the sigmoid colon in approximately 27 patients with a history of IBS [59]. However, transvaginal ultrasonography can only be performed in women and has limited acceptability.

Therefore, research on IBS diagnosis by ultrasound is insufficient. Several studies have been performed on the use of transvaginal and transrectal ultrasound [58–61]; however, these methods are painful and difficult to employ in home care settings and medical facilities.

In recent years, the percutaneous application of small ultrasound probes to the abdominal wall has made it possible to easily and noninvasively test for constipation [62]. In particular, elderly people with cognitive and motor dysfunction cannot complain of subjective symptoms, so it is important to be able to easily test for constipation and diarrhoea with compact ultrasonography devices. Ultrasound allows visualization of the interior of the body in real time and is non-invasive. Additionally, unlike abdominal X-ray, there is no exposure to radiation, and the test can be repeated. Furthermore, in recent years, the development of pocket-sized ultrasound devices has progressed rapidly [62], and ultrasound can be used in the examination room, at home, and at the bedside [63, 64].

Previous studies have reported that ultrasound can be used to objectively assess FC in elderly individuals who had ingested food; these studies classified the stool distribution pattern in the large intestine in patients with FC [62, 63]. A method involving the objective visualization of the large intestine is being developed for assessing constipation. Ultrasound has been used to visualize faecal retention in adults, including the elderly population, and Japanese healthcare professionals use ultrasound as an objective tool for constipation assessment, suggesting the feasibility of this method [64]. In addition, hyperechoic regions have been reported in the rectum of patients with FC [63–66]. A strong hyperechoic region with acoustic shadows in the rectum indicates the presence of hard stool and can be used to identify patients with IBS-C.

However, previous studies evaluated only patients with constipation and did not adequately screen patients with cognitive motor dysfunction or diarrhoea. In addition, ultrasound has traditionally been used to diagnose enteritis, an organic gastrointestinal disorder. There are specific, recognizable echoic findings that primarily indicate infectious enteritis and ischaemic colitis, and ultrasound can be used to diagnose these organic gastrointestinal disorders. It will be important to further investigate the utility of ultrasound as a non-invasive tool for diagnosing FGIDs.

In Japan, the 2016 revision of medical expenses [67] established a new “urination independence guidance fee” that requires a “residual urine measurement” via ultrasound and a “urination diary”. Previous studies developed an education program for nurses about ultrasound and suggested new evaluation methods [68–73]. If it becomes possible to diagnose FGIDs with ultrasound, it will be possible to individually tailor lifestyle and diet modifications and provide the necessary tools (incontinence pants, diapers, pads, etc.). In addition, drug administration decisions based on ultrasound results can make patients’ daily lives safer and easier. Therefore, it is important for future research to consider new assessment methods using ultrasound.

## Conclusions

FGIDs involve chronic or recurrent gastrointestinal symptoms. Laboratory tests do not indicate the presence of organic lesions, and the symptoms are due to dysfunction. IBS is a typical FGID. Defecation disorders in patients with IBS occur frequently and can have a significant negative impact on the daily life of patients, impacting their quality of life. The appropriate evaluation and analysis of colonic transit is important for resolving IBS-related defecation disorders. Diet and lifestyle factors also affect colonic transit; because changes in daily life affect colonic transit, it is necessary to properly assess patients’ lifestyles. A limitation of this study was the use of the subjective symptom-based Rome IV diagnostic criteria to diagnose IBS. This approach limits patient inclusion to those who can communicate and explain their symptoms. Colonic transit time and ultrasound are potential objective tools for IBS diagnosis. In particular, colonic transit was shown in some previous studies to be useful for accurately distinguishing between constipation and normal stool and for assessing delayed gastrointestinal motility. In the future, it will be necessary to consider additional methods for visualizing defecation disorders associated with gastrointestinal motility and objective indicators of abdominal symptoms associated with gastrointestinal motility abnormalities.

## Data Availability

As per the conditions outlined in the identifiable information included in the data file and survey materials, these items have not been made available.

## Abbreviations

IBS: Irritable Bowel Syndrome
FC: Functional Constipation
FGIDs: Functional Gastrointestinal Disorders
IBS-C: Irritable Bowel Syndrome with Constipation
NTC: Normal-Transit Constipation
STC: Slow-Transit Constipation

## References

[1] Longstreth GF, Thompson WG, Chey WD, Houghton LA, Mearin F, Spiller RC (2006). Functional bowel disorders. Gastroenterology..

[2] Drossman DA. Functional gastrointestinal disorders: history, pathophysiology, clinical features and Rome IV. Gastroenterology. 2016. 10.1053/j.gastro.2016.02.032. 10.1053/j.gastro.2016.02.032 27144617

[3] Drossman DA (2006). The functional gastrointestinal disorders and the Rome III process. Gastroenterology..

[4] Drossman DA, Hasler WL (2016). Rome IV-functional GI disorders: disorders of gut-brain interaction. Gastroenterology..

[5] Degen LP, Phillips SF (1996). How well does stool form reflect colonic transit?. Gut..

[6] Törnblom H, Van Oudenhove L, Sadik R, Abrahamsson H, Tack J, Simrén M (2012). Colonic transit time and IBS symptoms: what's the link?. Am J Gastroenterol.

[7] Ragnarsson G, Bodemar G (1999). Division of the irritable bowel syndrome into subgroups on the basis of daily recorded symptoms in two outpatients samples. Scand J Gastroenterol.

[8] Saad RJ, Rao SS, Koch KL, Kuo B, Parkman HP, McCallum RW (2010). Do stool form and frequency correlate with whole-gut and colonic transit? Results from a multicenter study in constipated individuals and healthy controls. Am J Gastroenterol.

[9] Burgell RE, Bhan C, Lunniss PJ, Scott SM (2012). Fecal incontinence in men: coexistent constipation and impact of rectal hyposensitivity. Dis Colon Rectum.

[10] Scarlett Y (2004). Medical management of fecal incontinence. Gastroenterology..

[11] Varma MG, Brown JS, Creasman JM, Thom DH, Van Den Eeden SK, Beattie MS (2006). Fecal incontinence in females older than aged 40 years: who is at risk?. Dis Colon Rectum.

[12] Fukudo S (2013). IBS: autonomic dysregulation in IBS. Nat Rev Gastroenterol Hepatol.

[13] Bouin M, Plourde V, Boivin M, Riberdy M, Lupien F, Laganière M (2002). Rectal distention testing in patients with irritable bowel syndrome: sensitivity, specificity, and predictive values of pain sensory thresholds. Gastroenterology..

[14] Kanazawa M, Palsson OS, Thiwan SI, Turner MJ, van Tilburg MA, Gangarosa LM (2008). Contributions of pain sensitivity and colonic motility to IBS symptom severity and predominant bowel habits. Am J Gastroenterol.

[15] Tack J, Müller-Lissner S, Stanghellini V, Boeckxstaens G, Kamm MA, Simren M (2011). Diagnosis and treatment of chronic constipation--a European perspective. Neurogastroenterol Motil.

[16] Lembo AJ, Schneier HA, Shiff SJ, Kurtz CB, MacDougall JE, Jia XD (2011). Two randomized trials of linaclotide for chronic constipation. N Engl J Med.

[17] Frattini JC, Nogueras JJ (2008). Slow transit constipation: a review of a colonic functional disorder. Clin Colon Rectal Surg.

[18] Bharucha AE, Pemberton JH, Locke GR (2013). American Gastroenterological Association technical review on constipation. Gastroenterology..

[19] Bouchoucha M, Devroede G, Bon C, Bejou B, Mary F, Benamouzig R (2017). Is-it possible to distinguish irritable bowel syndrome with constipation from functional constipation?. Tech Coloproctol.

[20] Spiller R, Aziz Q, Creed F, Emmanuel A, Houghton L, Hungin P (2007). Guidelines on the irritable bowel syndrome: mechanisms and practical management. Gut..

[21] Shah ED, Basseri RJ, Chong K, Pimentel M (2010). Abnormal breath testing in IBS: a meta-analysis. Dig Dis Sci.

[22] Pimentel M, Chow EJ, Lin HC (2003). Normalization of lactulose breath testing correlates with symptom improvement in irritable bowel syndrome. A double-blind, randomized, placebo-controlled study. Am J Gastroenterol.

[23] Cash BD, Schoenfeld P, Chey WD (2002). The utility of diagnostic tests in irritable bowel syndrome patients: a systematic review. Am J Gastroenterol.

[24] Lunniss PJ, Gladman MA, Benninga MA, Rao SS (2009). Pathophysiology of evacuation disorders. Neurogastroenterol Motil.

[25] Bharucha AE, Rao SS (2014). An update on anorectal disorders for gastroenterologists. Gastroenterology.

[26] Keller J, Layer P (2009). Intestinal and anorectal motility and functional disorders. Best Pract Res Clin Gastroenterol.

[27] Okawa Y (2021). A literature review of defecation care to prevent faecal incontinence in elderly individuals with irritable bowel syndrome. Jpn J Gastroenterol Hepatol.

[28] Scott SM, van den Berg MM, Benninga MA (2011). Rectal sensorimotor dysfunction in constipation. Best Pract Res Clin Gastroenterol.

[29] De Medici A, Badiali D, Corazziari E, Bausano G, Anzini F (1989). Rectal sensitivity in chronic constipation. Dig Dis Sci.

[30] Gladman MA, Aziz Q, Scott SM, Williams NS, Lunniss PJ (2009). Rectal hyposensitivity: pathophysiological mechanisms. Neurogastroenterol Motil.

[31] van der Sijp JR, Kamm MA, Nightingale JM, Britton KE, Mather SJ, Morris GP (1993). Radioisotope determination of regional colonic transit in severe constipation: comparison with radio opaque markers. Gut..

[32] Rao SS, Kuo B, McCallum RW, Chey WD, DiBaise JK, Hasler WL (2009). Investigation of colonic and whole-gut transit with wireless motility capsule and radiopaque markers in constipation. Clin Gastroenterol Hepatol.

[33] Hinton JM, Lennard-Jones JE, Young AC (1969). A NE method for studying gut transit times using radioopaque markers. Gut..

[34] Evans RC, Kamm MA, Hinton JM, Lennard-Jones JE (1992). The normal range and a simple diagram for recording whole gut transit time. Int J Color Dis.

[35] Arhan P, Devroede G, Jehannin B, Lanza M, Faverdin C, Dornic C (1981). Segmental colonic transit time. Dis Colon Rectum.

[36] Chaussade S, Khyari A, Roche H, Garret M, Gaudric M, Couturier D (1989). Determination of total and segmental colonic transit time in constipated patients. Results in 91 patients with a new simplified method. Dig Dis Sci.

[37] Kanazawa M, Endo Y, Whitehead WE, Kano M, Hongo M, Fukudo S (2004). Patients and nonconsulters with irritable bowel syndrome reporting a parental history of bowel problems have more impaired psychological distress. Dig Dis Sci.

[38] Koloski NA, Jones M, Kalantar J, Weltman M, Zaguirre J, Talley NJ (2012). The brain--gut pathway in functional gastrointestinal disorders is bidirectional: a 12-year prospective population-based study. Gut..

[39] Whitehead WE, Crowell MD, Robinson JC, Heller BR, Schuster MM (1992). Effects of stressful life events on bowel symptoms: subjects with irritable bowel syndrome compared with subjects without bowel dysfunction. Gut..

[40] Fukudo S, Suzuki J (1987). Colonic motility, autonomic function, and gastrointestinal hormones under psychological stress on irritable bowel syndrome. Tohoku J Exp Med.

[41] Welgan P, Meshkinpour H, Beeler M (1988). Effect of anger on colon motor and myoelectric activity in irritable bowel syndrome. Gastroenterology..

[42] Simrén M, Månsson A, Langkilde AM, Svedlund J, Abrahamsson H, Bengtsson U (2001). Food-related gastrointestinal symptoms in the irritable bowel syndrome. Digestion..

[43] Shinozaki M, Fukudo S, Hongo M, Shimosegawa T, Sasaki D, Matsueda K (2008). High prevalence of irritable bowel syndrome in medical outpatients in Japan. J Clin Gastroenterol.

[44] Cox SR, Prince AC, Myers CE, Irving PM, Lindsay JO, Lomer MC (2017). Fermentable carbohydrates [FODMAPs] exacerbate functional gastrointestinal symptoms in patients with inflammatory bowel disease: a randomised, double-blind, placebo-controlled, cross-over, re-challenge trial. J Crohns Colitis.

[45] Okawa Y, Fukudo S, Sanada H (2019). Specific foods can reduce symptoms of irritable bowel syndrome and functional constipation: a review. Biopsychosoc Med.

[46] Cozma-Petruţ A, Loghin F, Miere D, Dumitraşcu DL (2017). Diet in irritable bowel syndrome: what to recommend, not what to forbid to patients!. World J Gastroenterol.

[47] Bijkerk CJ, de Wit NJ, Muris JW, Whorwell PJ, Knottnerus JA, Hoes AW (2009). Soluble or insoluble fibre in irritable bowel syndrome in primary care? Randomised placebo controlled trial. BMJ.

[48] Bijkerk CJ, Muris JW, Knottnerus JA, Hoes AW, de Wit NJ (2004). Systematic review: the role of different types of fibre in the treatment of irritable bowel syndrome. Aliment Pharmacol Ther.

[49] Erdogan A, Rao SS, Thiruvaiyaru D, Lee YY, Coss Adame E, Valestin J (2016). Randomised clinical trial: mixed soluble/insoluble fibre vs. psyllium for chronic constipation. Aliment Pharmacol Ther.

[50] Khan MA, Nusrat S, Khan MI, Nawras A, Bielefeldt K (2014). Low-FODMAP diet for irritable bowel syndrome: is it ready for prime time?. Dig Dis Sci.

[51] Halmos EP, Power VA, Shepherd SJ, Gibson PR, Muir JG (2014). A diet low in FODMAPs reduces symptoms of irritable bowel syndrome. Gastroenterology.

[52] McIntosh K, Reed DE, Schneider T, Dang F, Keshteli AH, De Palma G (2017). FODMAPs alter symptoms and the metabolome of patients with IBS: a randomised controlled trial. Gut..

[53] Fukudo S, Kaneko H, Akiho H, Inamori M, Endo Y, Okumura T (2015). Evidence-based clinical practice guidelines for irritable bowel syndrome. J Gastroenterol.

[54] Zhao Y, Zou D, Wang R, Ma X, Yan X, Man X (2010). Dyspepsia and irritable bowel syndrome in China: a population-based endoscopy study of prevalence and impact. Aliment Pharmacol Ther.

[55] Guliter S, Yilmaz S, Yakaryilmaz F, Keles H (2005). Evaluation of gallbladder motility in patients with irritable bowel syndrome. Swiss Med Wkly.

[56] Güçlü M, Pourbagher A, Serin E, Kul K, Ozer B, Cosar A (2006). Ultrasonographic evaluation of gallbladder functions in patients with irritable bowel syndrome. J Gastroenterol Hepatol.

[57] Kusunoki H, Kamada T, Sato M, Haruma K, Hata J (2006). Ultrasonographic assessment of sigmoid colon in patients with irritable bowel syndrome. Nihon Rinsho.

[58] Devanarayana NM, Rajindrajith S, Bandara C, Shashiprabha G, Benninga MA (2013). Ultrasonographic assessment of liquid gastric emptying and antral motility according to the subtypes of irritable bowel syndrome in children. J Pediatr Gastroenterol Nutr.

[59] Crade M, Pham V (2006). Ultrasound examination of the sigmoid colon: possible new diagnostic tool for irritable bowel syndrome. Ultrasound Obstet Gynecol.

[60] Awad RA (1998). Martin J, Cal y major M, Noguera JL, Ramos R, Amezcua C, et al. Transrectal ultrasonography: relationship with anorectal manometry, electromyography and sensitivity tests in irritable bowel syndrome. Int J Color Dis.

[61] O'Connor OJ, McSweeney SE, McWilliams S, O'Neill S, Shanahan F, Quigley EM (2012). Role of radiologic imaging in irritable bowel syndrome: evidence-based review. Radiology..

[62] Yabunaka K, Matsumoto M, Yoshida M, Tanaka S, Miura Y, Tsutaoka T (2018). Assessment of rectal feces storage condition by a point-of-care pocket-size ultrasound device for healthy adult subjects: a preliminary study. Drug Discov Ther.

[63] Tanaka S, Yabunaka K, Matsumoto M, Tamai N, Noguchi H, Yoshida M (2018). Fecal distribution changes using colorectal ultrasonography in older people with physical and cognitive impairment living in long-term care facilities: a longitudinal observational study. Healthcare (Basel).

[64] Matsumoto M, Tanaka S, Yabunaka K, Yoshida M, Miura Y, Tsutaoka T (2018). Ultrasonographic evaluation of changes over time in one defecation cycle in adults with functional constipation: a report of two cases. Drug Discov Ther.

[65] Yabunaka K, Nakagami G, Tabata K, Sugama J, Matsumoto M, Kido Y (2018). Constipation in the elderly in a Japanese long-term medical facility: an ultrasonographic investigation. Drug Discov Ther.

[66] Okawa Y (2020). Can irritable bowel syndrome be detected by ultrasound?. Drug Discov Ther.

[67] Ministry of Health, Labor and Welfare Insurance Bureau. Outline of Medical Fee Revision in 2016. Japanese Version. [JPN].

[68] Yamada T, Minami T, Soni NJ, Hiraoka E, Takahashi H, Okubo T (2018). Skills acquisition for novice learners after a point-of-care ultrasound course: does clinical rank matter?. BMC Med Educ.

[69] Selim AA, Ramadan FH, El-Gueneidy MM, Gaafer MM (2012). Using objective structured clinical examination (OSCE) in undergraduate psychiatric nursing education: is it reliable and valid?. Nurse Educ Today.

[70] Smith V, Muldoon K, Biesty L (2012). The objective structured clinical examination (OSCE) as a strategy for assessing clinical competence in midwifery education in Ireland: a critical review. Nurse Educ Pract.

[71] Cawthorn TR, Nickel C, O'Reilly M, Kafka H, Tam JW, Jackson LC (2014). Development and evaluation of methodologies for teaching focused cardiac ultrasound skills to medical students. J Am Soc Echocardiogr.

[72] Duff B, Massey D, Gooch R, Wallis M (2018). The impact of a multimodal education strategy (the DeTER program) on nurses' recognition and response to deteriorating patients. Nurse Educ Pract.

[73] Wanjiku GW, Bell G, Wachira B (2018). Assessing a novel point-of-care ultrasound training program for rural healthcare providers in Kenya. BMC Health Serv Res.

